# Cloning and Functional Characterization of Two 4-Coumarate: CoA Ligase Genes from *Selaginella moellendorffii*

**DOI:** 10.3390/molecules23030595

**Published:** 2018-03-07

**Authors:** Xin-Yan Liu, Ping-Ping Wang, Yi-Feng Wu, Ai-Xia Cheng, Hong-Xiang Lou

**Affiliations:** Key Laboratory of Chemical Biology of Natural Products, Ministry of Education, School of Pharmaceutical Sciences, Shandong University, Jinan 250012, China; qqbailiu@163.com (X.-Y.L.); wangpingp1234@163.com (P.-P.W.); wendywu.12@163.com (Y.-F.W.)

**Keywords:** *Selaginella moellendorffii*, 4-coumarate: CoA ligase, biochemical characterization

## Abstract

*Selaginella* is an extant lycopodiophyte genus, which is representative of an ancient lineage of tracheophytes. The important evolutionary status makes it a valuable resource for the study of metabolic evolution in vascular plants. 4-coumarate: CoA ligase (4CL) is the pivotal enzyme that controls the flow of carbon through the phenylpropanoid metabolic pathway into the specific lignin, flavonoid, and wall-bound phenolics biosynthesis pathways. Although 4CLs have been extensively characterized in other vascular plants, little is known of their functions in *Selaginella*. Here, we isolated two *4CL* genes (*Sm4CL1* and *Sm4CL2*) from *Selaginella moellendorffii*. Based on the enzymatic activities of the recombinant proteins, both of these genes encoded bona fide 4CLs. The 4CL isoforms in *S. moellendorffii* have different activities: Sm4CL2 was more active than Sm4CL1. The enzymatic properties and gene expression patterns indicated that the *4CL* genes have been conserved in the evolution of vascular plants.

## 1. Introduction

**Figure 1:**
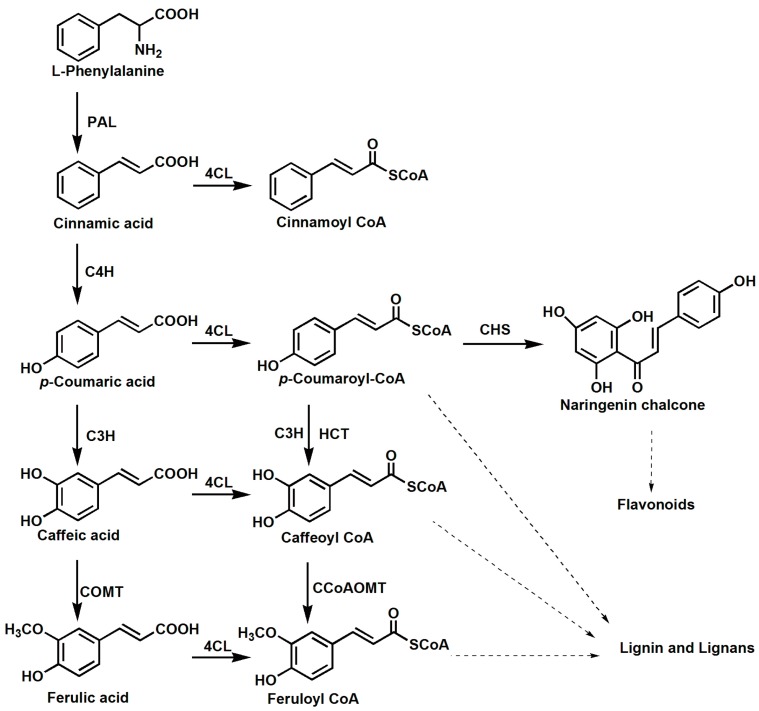
The proposed synthesis pathway of phenylpropanoids in *Selaginella moellendorffii*. PAL: phenylalanine ammonia lyase, C4H: cinnamate 4-hydroxylase, 4CL: 4-coumarate: CoA ligase, C3H: 4-coumarate 3-hydroxylase, COMT: caffeic acid *O*-methyl transferase, HCT: *p*-hydroxy cinnamoyl transferase, CCoAOMT: caffeoyl-CoA *O*-methyltransferase, CHS: chalcone synthase.

Phenylpropanoids are plant-specific natural products that serve important functions during growth, development, and environmental interactions [1]. Phenylpropanoids are synthesized from phenylalanine via the central phenylpropanoid pathway, which is mediated by phenylalanine ammonia-lyase (PAL), cinnamate 4-hydroxylase (C4H), and 4-coumarate: CoA ligase (4CL). 4-coumarate: CoA ligase (EC 6.2.1.12) catalyzes the conversion of several hydroxycinnamic acids into their corresponding CoA esters, enabling the biosynthesis of a diverse array of natural products (Figure 1), including lignin, flavonoids, and wall-bound phenolics [2,3,4]. The multiple functions of 4CL might explain why it is encoded by a gene family present in plants. The *4CL* gene family has four members in *Arabidopsis thaliana* [5], five members in *Oryza sativa* [6], and four members in *Physcomitrella patens* [7]. The catalytic properties of 4CL protein isoforms have been studied extensively. In some species, such as potato and parsley, cloned genes encode identical, or nearly identical, proteins that possess similar substrate affinities [8,9]. In other plants, such as aspen trees and *Arabidopsis*, structurally- and functionally-divergent protein isozymes have been isolated [5,10].

*Selaginella*, also known as spikemoss, is the only surviving genus within the Selaginellaceae. This genus first appears in the fossil record some 400 million years ago. Selaginellaceae, together with two other extant families (Lycopodiaceae, the clubmosses, and Isoetaceae, the quillworts), comprise the division Lycopodiophyta, the oldest extant lineage of vascular plants [11]. Over the last several decades, the isolation and structural elucidation of phenylpropanoids from the *Selaginella* genus has expanded. In vascular plants, deposition of lignin is of significance because it reinforces plant cell walls, facilitates water transport, provides compressive strength to conducting tissues, and acts as a mechanical barrier to pathogens. Additionally, previous investigations revealed the genus *Selaginella* to be a rich source of flavonoids, including monomeric flavonoids and biflavonoids [12]. However, little is known about the genes in the phenylpropanoid pathways of *Selaginella* species. Recently, the genome of *S. moellendorffii* was sequenced by the Joint Genome Institute (JGI) because of its important evolutionary position among land plants and its small genome size; *S. moellendorffii* is, therefore, an important new model species [13,14]. Here, we isolated and identified two *4CL* genes (*Sm4CL1* and *Sm4CL2*) from *S. moellendorffii*. The activities of these two enzymes were substantially different. We also measured the organizational expression patterns of the two genes.

## 2. Results

### 2.1. Identification of 4CL Genes from S. moellendorffii

**Figure 2:**
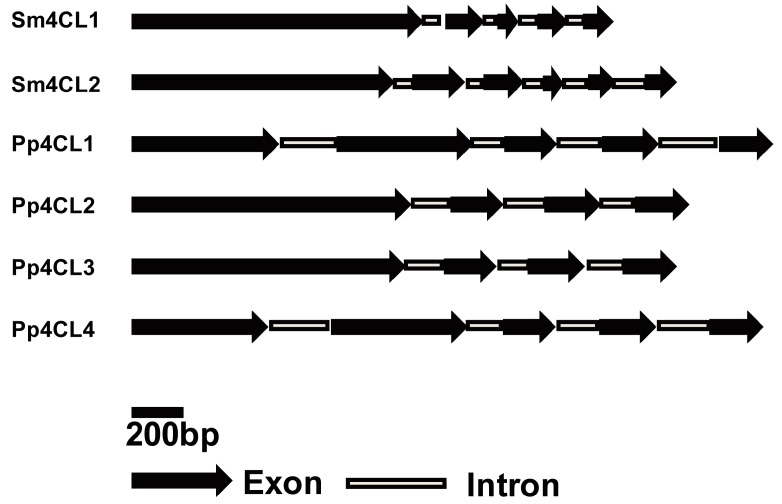
Genome structure of *Sm4CLs* and other plant *4CL* genes. Schematic diagrams with exons (black arrow) and introns (gray box). The gene accession numbers are as follows: *Pp4CL1* (*Physcomitrella patens*): EU167552; *Pp4CL2* (*P. patens*): EU167553; *Pp4CL3* (*P. patens*): EU167554; *Pp4CL4* (*P. patens*): EU167555.

We identified and cloned two putative *4CL* genes (*Sm4CL1*: GenBank accession number XP_002969881; *Sm4CL2*: XP_002979073) from the *S. moellendorffii* genome. The ORF of *Sm4CL1* was 1614 bp, predicting a protein of 537 residues, with a molecular mass of 57.34 kDa and a pI of 5.88. The ORF of *Sm4CL2* was 1695 bp, predicting a protein of 564 residues, with a molecular mass of 61.56 kDa and a pI of 5.87. *Sm4CL1* contained five exons and four introns, while *Sm4CL2* contained six exons and five introns (Figure 2). *Sm4CL* genes had multiple introns and exons, consistent with *4CL* genes in other species [7].

### 2.2. Protein Sequence and Phylogenetic Analysis of Sm4CLs

**Figure 3:**
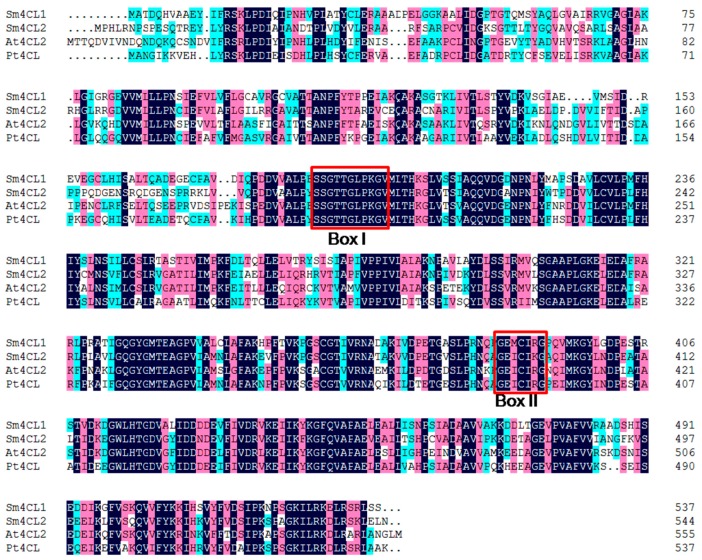
Sequence alignment of Sm4CLs with other plant 4CLs. Box I represents the putative AMP-binding domain, and box II represents the conserved “GEICIGR” putative catalytic site. Sequence accession numbers are given in Supplementary Data Table S2.

The predicted proteins Sm4CL1 and Sm4CL2 had 55–60% sequence identity with 4CL proteins in other species of plants (At4CL2 and Pt4CL). Our multiple sequence alignment indicated that both Sm4CLs contained two motifs that are conserved across the 4CL family in plants: a putative AMP-binding motif (box I) and a conserved box II “GEICIRG” domain (Figure 3). The central cysteine residue of box II might be involved in catalysis [15].

**Figure 4:**
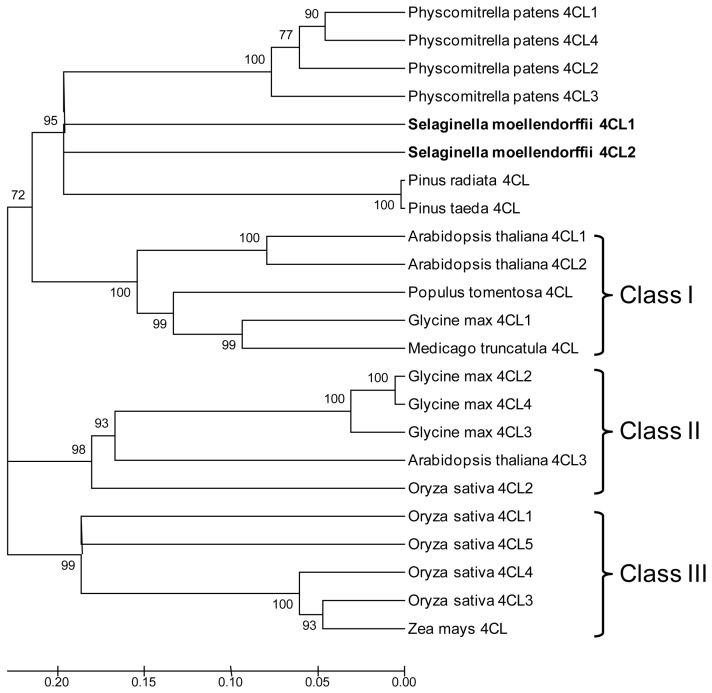
Neighbor-joining phylogeny of Sm4CLs and previously published 4CL gene sequences from other species. Sequence accession numbers are given in Supplementary Data Table S2.

Our neighbor-joining phylogenetic analysis divided the 4CL proteins into four clades: one clade of Sm4CL1, Sm4CL2, and 4CL proteins from mosses and gymnosperms; and three angiosperm 4CL clades (class I, class II, and class III) (Figure 4). Angiosperm class I 4CLs are more closely associated with lignin biosynthesis, as demonstrated by antisense and RNAi suppression studies [6,16,17]. Angiosperm class II 4CLs are likely involved the biosynthesis of other phenolic compounds, based on their compartmentalized expression in various plant organs and tissues [10,18]. Angiosperm class I and II 4CLs are predominantly present in dicots, whereas class III 4CLs are largely restricted to monocots.

### 2.3. Biochemical Characterization of Recombinant Sm4CL In Vitro

**Figure 5:**
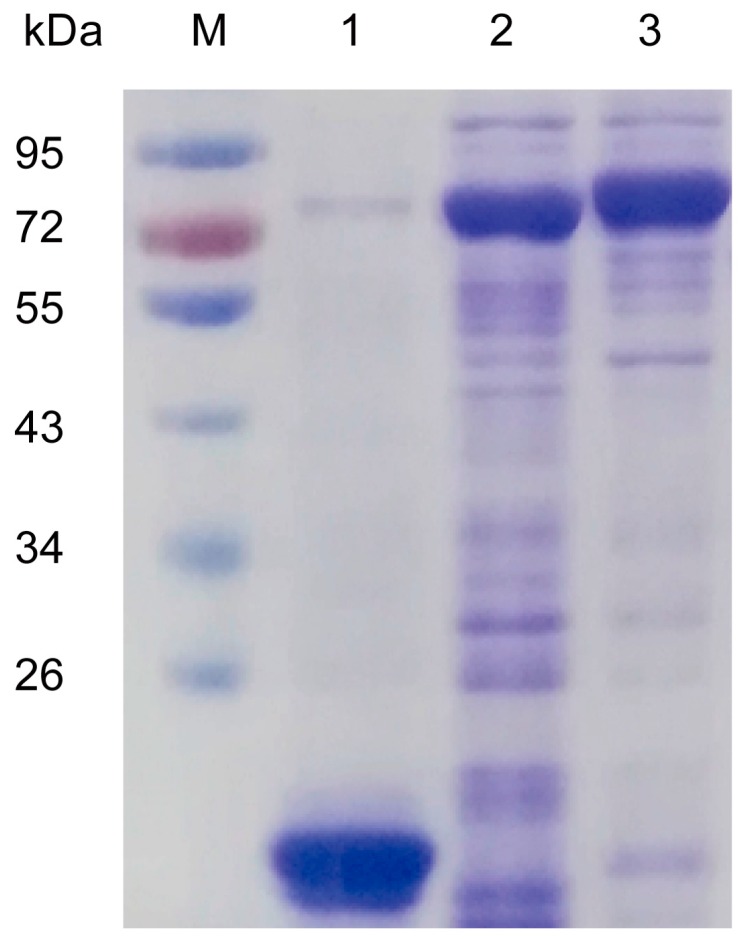
SDS-PAGE analysis of purified recombinant proteins from *E. coli*. Lane M: molecular mass standards; lane 1: empty pET32a vector; lane 2: pET32a-Sm4CL1; lane 3: pET32a-Sm4CL2.

**Figure 6:**
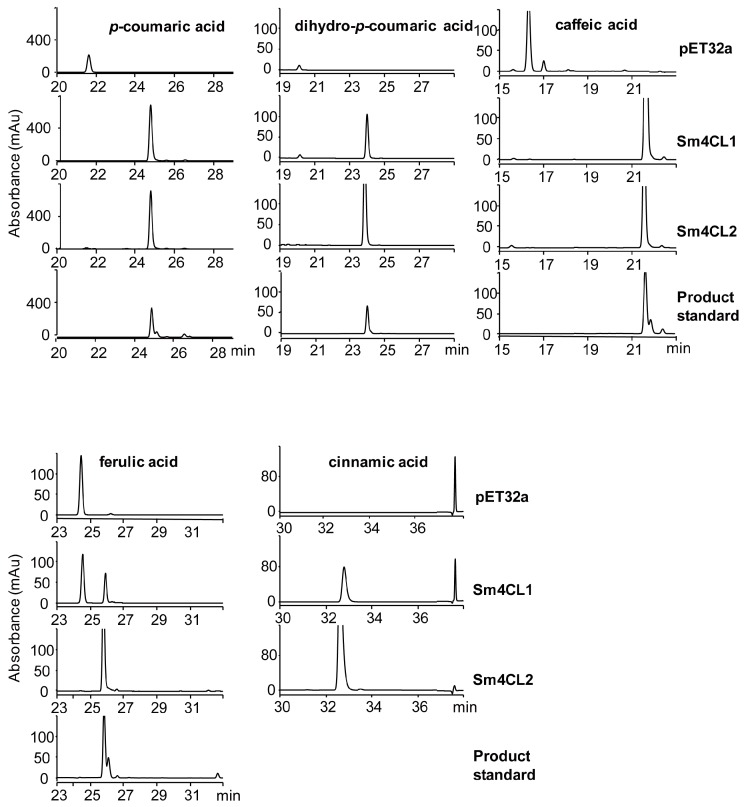
HPLC profiles of reaction products generated by recombinant Sm4CLs. Recombinant Sm4CL enzymes were provided with *p*-coumaric acid, dihydro-*p*-coumaric acid, caffeic acid, ferulic acid, and cinnamic acid as substrates. We used a bacterial clone inoculated with an empty pET32a vector to produce the control reaction product.

We examined the biochemical properties of the Sm4CLs by synthesizing these enzymes in *Escherichia coli*. Following purification of the corresponding recombinant 4CLs, fractionation with sodium dodecyl sulfate polyacrylamide gel electrophoresis (SDS-PAGE) showed that the molecular masses were between 75 and 79 kDa (including the tags) (Figure 5). We tested whether the purified proteins utilized *p*-coumaric acid, dihydro-*p*-coumaric acid, *trans*-cinnamic acid, caffeic acid, ferulic acid, and sinapic acid. Both Sm4CL1 and Sm4CL2 had distinct CoA ligation activity with all substrates except sinapic acid (Table 1). Reaction products were identified by HPLC using *p*-coumaroyl CoA, dihydro-*p*-coumaroyl CoA, caffeoyl CoA, and feruloyl CoA as product standards (Figure 6). We determined the effects of pH and temperature on the enzyme activity with *p*-coumaric acid as substrate. The enzymatic activities of the Sm4CLs were pH-dependent and temperature-sensitive. The optimum pH for both Sm4CL1 and Sm4CL2 was about 7.0. The optimum temperature for Sm4CL1 was 40 °C, while for Sm4CL2, it was 50 °C. Under optimal conditions, recombinant Sm4CL1 showed a high affinity for *p*-coumaric acid (Km = 11.89 μM) and caffeic acid (Km = 10.87 μM) (Table 2). However, the substrate turnover rate (kcat) was higher with *p*-coumaric acid than with caffeic acid, resulting in a higher catalytic efficiency (kenz) with *p*-coumaric acid. Thus, *p*-coumaric acid was the best substrate for Sm4CL1.

Similarly, Sm4CL2 showed a higher affinity for *p*-coumaric acid (Km = 19.67 μM) and caffeic acid (Km = 18.96 μM), and a higher substrate turnover rate with *p*-coumaric acid, ferulic acid, and dihydro-*p*-coumaric acid (Table 2). Based on the Km value and the substrate turnover rate, we considered *p*-coumaric acid the best substrate for Sm4CL2. 

We also found that the turnover rate of Sm4CL2 was much higher than that of Sm4CL1: the kcat of *p*-coumaric acid for Sm4CL2 was 42.91 min^−1^, and while that of Sm4CL1 was only 4.09 min^−1^.

### 2.4. Determination of Gene Expression Patterns in Tissues

**Figure 7:**
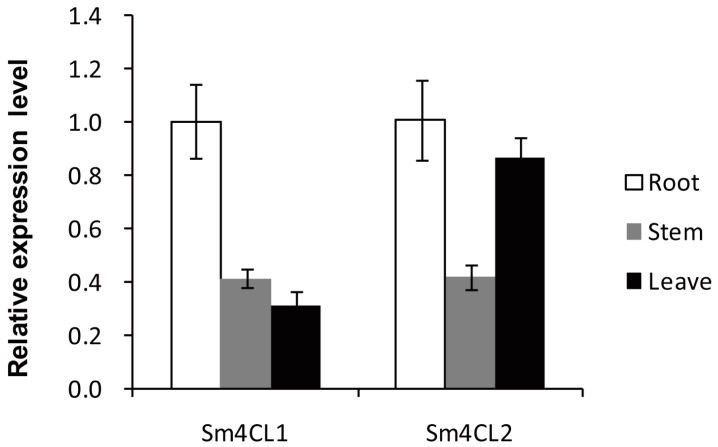
Expression of *Sm4CL* genes in roots, stems, and leaves of *S. moellendorffii*. Values represent the mean ± SD, measured from at least three biological replicates.

Transcription of *Sm4CL1*, *Sm4CL2* was detected in roots, stems and leaves, but the expression level of the two genes was different. *Sm4CL1* was most highly expressed in roots, then stems, then leaves. *Sm4CL2* was also the most highly expressed in roots like *Sm4CL1*, but its expression level in the leaves was higher than in the stems (Figure 7).

## 3. Discussion

*Selaginella* species are evolutionarily important among land plants: these are the oldest vascular plants and are distinguished from primitive bryophytes by the development of vascular tissue capable of transporting fluids throughout the plant body. 4CL is the pivotal enzyme that controls the flow of carbon through the phenylpropanoid metabolic pathway into the specific lignin, flavonoid, and coumarin biosynthesis pathways. The *4CL* genes have been widely investigated in other lineages of vascular plants, but little is known about *4CL* genes in *Selaginella*. A better understanding of the *4CL* genes in *Selaginella* will inform our understanding of the evolution of *4CLs* across all vascular plants.

We cloned two *4CL* genes from *S. moellendorffii*, based on the enzymatic activities of the recombinant proteins, both of these genes encoded bona fide 4CLs. This was consistent with previous reports that the *4CL* genes exist as a gene family in most vascular plants [10,19,20,21]. We noticed that the properties of the *Sm4CL* genes we investigated were similar to those of *4CLs* in other vascular plants: their genetic structure was made up of multiple introns and exons, they contained conserved putative catalytic domains, and they had close phylogenetic relationships with other vascular plant 4CLs. *Sm4CL1* and *Sm4CL2* were more highly expressed in the roots than in the stems or the leaves; this is consistent with previous studies [22,23,24]. These results indicated the *4CL* genes are evolutionarily conserved, underlining the importance of 4CL as a key enzyme in the phenylpropanoid metabolism of all vascular plants.

Although the Sm4CL1 and Sm4CL2 isoforms clustered together in our phylogeny, the activities of these two enzymes were substantially different. The catalytic efficiency of Sm4CL2 toward hydroxycinnamic acids was similar to that of other plant 4CLs [22,23,25,26], but the enzymatic activity of Sm4CL1 toward hydroxycinnamic acids was weak. Our kinetic analysis indicated that Sm4CL2 efficiently catalyzed the conversions of *p*-coumaric acid, caffeic acid, and ferulic acid into their corresponding CoA esters. Meanwhile, lignin in *Selaginella* species is comprised of three major types of aromatic units: *p*-hydroxyphenyl (H), guaiacyl (G), and syringyl (S), which are derived from *p*-coumaryl alcohol, coniferyl alcohol, and sinapyl alcohol, respectively [3,27,28,29,30,31]. Therefore, it is likely that Sm4CL2 plays a role in the biosynthesis of the *p*-hydroxyphenyl and guaiacyl lignin subunits. The lack of any detectable conversion of sinapate by each Sm4CL isoforms, and the existence of ferulate 5-hydroxylation and *O*-methylation pathway which operates on feruloyl-CoA to produce sinapoyl-CoA [32,33], suggested that the biosynthesis of sinapyl alcohol and syringyl lignin occurs via an independent 4CL pathway in *S. moellendorffii*.

In summary, we identified two *4CL* genes from the *S. moellendorffii* genome. These genes were expressed in all tested organs. Our bioinformatic characterization and *in vitro* enzyme assay indicated that typical *4CL* genes were evolutionarily conserved across the vascular plants. The enzyme activities of Sm4CLs were interesting, which included a highly-active enzyme and a very weakly active enzyme. This paper provides a framework for future work on the phenylpropanoid metabolic pathway in *S. moellendorffii*.

## 4. Materials and Methods

### 4.1. Plant Material, Nucleic Acid Extraction, and Reagents 

*S. moellendorffii* was grown in a greenhouse under controlled conditions: 25 °C with a 12 h photoperiod. Genomic DNA was extracted from fresh ferny foliage using the CTAB method [34]. Total RNA was extracted from foliage using the modified CTAB method [35], and was used as a template for cDNA synthesis using PrimeScript RT Master Mix (Takara, Otsu, Japan), following the manufacturer’s protocols. All chemicals and reagents were purchased from Sigma-Aldrich (St. Louis, MO, USA), unless otherwise indicated. *p*-Coumaroyl CoA, dihydro-*p*-coumaroyl CoA, caffeoyl CoA, and feruloyl CoA were enzymatically synthesized following previously-published procedures [36,37,38].

### 4.2. DNA Isolation and Sequence Analysis

We identified two putative *4CL* genes in the genome of *S. moellendorffii* v.1.0 (http://phytozome.jgi.doe.gov/pz/portal.html) [39] using the TblastN algorithm and designated these putative genes *Sm4CL1* and *Sm4CL2*. The full-length cDNA sequences of these genes were amplified using the corresponding primer pairs (Sm4CL1-F/R, Sm4CL2-F/R; Supplementary Data Table S1). The *Sm4CL* genomic DNA sequences were also obtained from genomic DNA with the same corresponding primer pairs (Supplementary Data Table S1). Predicted polypeptide sequences were aligned using DNAMAN v.7.0.2 (Lynnon Corp., Vaudreuil-Dorion, QC, Canada). Neighbor-joining phylogenetic trees were constructed using MEGA v5.0 [40]. We tested tree robustness by running 1000 bootstrap replicates.

### 4.3. Recombinant Protein Expression and Purification

The open reading frames (ORFs) of *Sm4CL* were amplified with pET primer pairs (Table S1), and subcloned into a pET32a vector. After the sequences were confirmed, each construct was transformed into *Escherichia coli* strain BL21 (DE3) for heterologous expression. The transgenic cultures were incubated at 37 °C until the OD_600_ reached 0.4–0.6, then the recombinant proteins were induced at 16 °C for 16 h after adding 0.5 mM isopropyl β-d-1-thiogalactopyranoside (IPTG). N-terminal hexahistidine-tagged proteins were purified by passing through a Ni-NTA Sefinose His-bind column (Bio Basic Inc., Markham, ON, Canada), and then were exchanged through an Ultrafiltration tube (Millipore, Billerica, MA, USA) in the presence of binding buffer (20 mM Tris–HCl, 500 mM NaCl, pH 8.0). Protein concentrations were determined with the Bradford reagent (Beyotime, Shanghai, China), using BSA as a standard. The resulting purified proteins were monitored on SDS-PAGE, using Coomassie Blue R250 staining.  The migration of standard molecular weight markers (10–170 kDa) (Fermentas, Waltham, MA, USA) was used to estimate the molecular masses of the target proteins.

### 4.4. Enzyme Assays

We performed Sm4CLs enzyme assays to detect the formation of the CoA esters of various cinnamic acid derivatives. Each 200 μL assay contained 10 μg purified protein, 200 μM substrate, 5 mM ATP, 300 μM CoA, and 5 mM MgCl_2,_ made up in 200 mM Tris-HCl buffer (pH 7.5). Enzymatic reactions were incubated for 30 min at 30 °C, and the reaction products were analyzed using a HPLC device (1260 Infinity Binary LC system, Agilent, Santa Clara, CA, USA), equipped with a multi wavelength detector. The samples were separated through a 5-μm reverse-phase XDB-C18 column with a flow rate of 1 mL/min. A linear gradient of solvent A (1% H_3_PO_4_ in H_2_O) and solvent B (CH_3_CN) were applied as follows: 0–5 min, 5% B isocratic; 5–35 min, 5–25% B linear; 35–36 min, 25–100% B linear. Standard solutions of reference compounds were used for calibration.

The effects of pH and temperature on the enzyme activity were examined using *p*-coumaric acid as substrate. To determine optimal pH, enzymatic activity was assessed in 200 mM Tris-HCl buffer (pH 5.0, 6.0, 6.5, 7.0, 7.5, 8.0, 8.5, and 9.0), while the optimal temperature was determined by measuring enzymatic activity at 10, 20, 30, 40, 50, and 60 °C. All experiments were performed in triplicate. Kinetic parameters were determined using different substrate concentrations. We performed this experiment in triplicate, with 3 μg purified enzyme in a final volume of 200 μL of 200 mM Tris-HCl buffer at the optimal pH and temperature for 10 min. The level of UV absorption was recorded at 1 min intervals. Relevant wavelengths were 311 nm (cinnamoyl CoA), 333 nm (*p*-coumaroyl CoA), 346 nm (caffeoyl CoA), 346 nm (feruloyl CoA), and 352 nm (sinapoyl CoA) [37,41,42]. For determining kinetic properties with dihydro-*p*-coumaric acid, reactions were incubated at optimal temperature for 10 min and analyzed using HPLC, and then the quantity of the reaction product present was estimated from a standard calibration curve.

### 4.5. Expression Profile Analysis

For real-time quantitative PCR, various *S. moellendorffii* tissues (roots, stems, and leaves) were collected and snap-frozen in liquid N_2_. Total RNA was extracted and cDNA was synthesized as described above. Transcript abundance was quantified using real-time quantitative PCR based on the corresponding RT primer pairs (sequences given in Table S1), using an Eppendorf Mastercycler ep realplex RealTime PCR System (Eppendorf, Hamburg, Germany). Each 10 μL reaction contained 1 μg cDNA, 0.5 μM of each primer, and 1 × SYBR Green PCR Master mix. A fragment of the *S. moellendorffii* geneome encoding an actin gene was used as the internal reference sequence and was amplified using primers Smactin-RTF/R (Table S1).

## Figures and Tables

**Table 1:** The substrate specificity of Sm4CLs.

Substrate	Specific Activity (nmol mg^−1^ min^−1^)	
	Sm4CL1	Sm4CL2
*p*-coumaric acid	71.77 ± 0.31	557.5 ± 15.58
caffeic acid	16.92 ± 0.2	239.71 ± 5.6
cinnamic acid	2.29 ± 0.08	283.08 ± 5.22
ferulic acid	1.14 ± 0.00	494.83 ± 3.61
dihydro-*p*-coumaric acid	16.71 ± 0.45	271.77 ± 29.57
sinapic acid	ND	ND

ND: No detectable activity.

**Table 2:** Kinetic parameters of Sm4CLs.

Enzyme	Substrate	Km (μM)	Vmax (nmol mg^−1^ min^−1^)	kcat (min^−1^)	kenz (M^−1^ min^−1^)
Sm4CL1	*p*-coumaric acid	11.89 ± 1.56	71.37 ± 2.58	4.09 ± 0.15	343,986.54
	caffeic acid	10.87 ± 1.6	25.40 ± 0.94	1.46 ± 0.05	134,314.63
	di-*p*-coumaric acid	442.4 ± 53.74	43.73 ± 1.34	2.51 ± 0.08	5673.6
Sm4CL2	*p*-coumaric acid	19.67 ± 2.92	697.1 ± 32.06	42.91 ± 1.97	2,181,494.66
	caffeic acid	18.96 ± 2.61	268.1 ± 9.74	16.50 ± 0.60	870,253.16
	cinnamic acid	126.9 ± 11.57	382 ± 9.31	23.52 ± 0.57	185,310.64
	ferulic acid	95.99 ± 13.39	525.2 ± 21.18	32.33 ± 1.3	336,819.59
	di-*p*-coumaric acid	220.1 ± 36.86	673.1 ± 23.19	41.44 ± 1.43	188,278.06

## Supplementary Materials

The following are available online. Table S1: Primer sequences used in this research, Table S2: Accession numbers of amino acid sequences used for phylogenetic reconstruction.
