# Supplementary material for: Cloning and Functional Characterization of Two 4-Coumarate: CoA Ligase Genes from Selaginella moellendorffii
Source: Molecules. 2018 Mar 7;23(3):595. doi: 10.3390/molecules23030595 (PMC6017068; doi:10.3390/molecules23030595)
Supplement: Supplementary file 1 [file molecules-23-00595-s001.pdf]

Table S1

Primer sequences used in this research.

Primer name	Primer sequences (5'-3')
Sm4CL1-F	ATGGCGACGGATCAGCATGT
Sm4CL1-R	CTAAGAGGAAAGTCTACTTC
Sm4CL2-F	ATGCCACATTTGCGCAATCC
Sm4CL2-R	TTAGTTTAGCTCCAGCTTGC
Sm4CL1-pETF	CGGGATCCATGGCGACGGATCAGCATGT
Sm4CL1-pETR	CCAAGCTTCTAAGAGGAAAGTCTACTTC
Sm4CL2-pETF	CGCGATATCATGCCACATTTGCGCAATCC
Sm4CL2-pETR	ATAAGAATGCGGCCGCTTAGTTTAGCTCCAGCTTGC
Sm4CL1-RTF	GAGGTGTTCATTGTGGACCG
Sm4CL1-RTR	TTGGAGACGAAGCCCTTGAT
Sm4CL2-RTF	AACCCGATCGTGGACAAGTA
Sm4CL2-RTR	CATACCCCTGACCGAGTACC
Smactin-RTF	ACTGGGACGACATGGAGAAG
Smactin-RTR	CCGCCTGAATAGCAACGTAC

Table S2

Accession numbers of amino acid sequences used for phylogenetic reconstruction.

Sequence	Accession No.
Physcomitrella patens 4CL1	ABY21312

Physcomitrella patens 4CL4	ABY21315
Physcomitrella patens 4CL2	ABY21313
Physcomitrella patens 4CL3	ABY21314
Pinus radiate 4CL	ACF35279
Pinus taeda 4CL	AAB42383
Arabidopsis thaliana 4CL1	NP_001077697
Arabidopsis thaliana 4CL2	NP_188761
Populus tomentosa 4CL	AAL02144
Glycine max 4CL1	NP_001236418
Medicago truncatula 4CL	XP_003637266
Glycine max 4CL2	NP_001236236
Glycine max 4CL3	AAC97389
Glycine max 4CL4	CAC36095
Arabidopsis thaliana 4CL3	NP_849844
Oryza sativa 4CL1	XP_015650724
Oryza sativa 4CL2	XP_015624111
Oryza sativa 4CL3	XP_015625716
Oryza sativa 4CL4	XP_015643415
Oryza sativa 4CL5	XP_015650830
Zea mays 4CL	AAS67644
